# SNP and gene networks construction and analysis from classification of copy number variations data

**DOI:** 10.1186/1471-2105-12-S5-S4

**Published:** 2011-07-27

**Authors:** Yang Liu, Yiu Fai Lee, Michael K Ng

**Affiliations:** 1 Department of Mathematics, Hong Kong Baptist University, Kowloon Tong, Hong Kong

## Abstract

**Background:**

Detection of genomic DNA copy number variations (CNVs) can provide a complete and more comprehensive view of human disease. It is interesting to identify and represent relevant CNVs from a genome-wide data due to high data volume and the complexity of interactions.

**Results:**

In this paper, we incorporate the DNA copy number variation data derived from SNP arrays into a computational shrunken model and formalize the detection of copy number variations as a case-control classification problem. More than 80% accuracy can be obtained using our classification model and by shrinkage, the number of relevant CNVs to disease can be determined. In order to understand relevant CNVs, we study their corresponding SNPs in the genome and a statistical software PLINK is employed to compute the pair-wise SNP-SNP interactions, and identify SNP networks based on their *P*-values. Our selected SNP networks are statistically significant compared with random SNP networks and play a role in the biological process. For the unique genes that those SNPs are located in, a gene-gene similarity value is computed using GOSemSim and gene pairs that have similarity values being greater than a threshold are selected to construct gene networks. A gene enrichment analysis show that our gene networks are functionally important.

Experimental results demonstrate that our selected SNP and gene networks based on the selected CNVs contain some functional relationships directly or indirectly to disease study.

**Conclusions:**

Two datasets are given to demonstrate the effectiveness of the introduced method. Some statistical and biological analysis show that this shrunken classification model is effective in identifying CNVs from genome-wide data and our proposed framework has a potential to become a useful analysis tool for SNP data sets.

## Background

Copy number variation (CNV) is defined as a genomic segment range from one kilobase to several megabases in size, in which copy number differences have been observed by comparison of two or more reference genomes [1,2]. Human beings ordinarily have two copies of each autosomal region, one per chromosome. CNVs can be caused by genomic rearrangements such as inversions, deletions and duplications.

The fact that DNA copy number variation is a widespread and common phenomenon among human beings was first discovered [3,4] following the completion of the human genome project. The high variability of the copy number throughout the human genome have been found by investigating on 270 HapMap individuals [5,6]. Various studies have been developed for genome-wide CNV analysis to suggest their significant role in understanding human genetics. Redon et al. [7] published the first comprehensive and global map of CNVs in a genome-wide scale for making the beginning of large-scale copy number analysis. Comparative genomic hybridization (CGH) [8] and array-based comparative genomic hybridization [9] can detect gains and losses of genomic segments. Based on CGH, Wang et al. [10] proposed a new algorithm “Cluster Along Chromosomes” (CLAC), which builds hierarchical clustering-style trees along each chromosome and selects the interesting clusters by controlling False Discovery Rate (FDR) at a certain level. Bayesian model is a popular method for CNV detection. Pique-Regi et al. [11] exploited the use of piecewise constant (PWC) vectors to represent genome copy number and sparse Bayesian learning (SBL) to detect CNA breakpoints. Wu et al. [12] implemented Bayesian segmentation approach to carry out segmentation and assigning copy number status simultaneously. Recently, Chen et al. [13] used the mean and variance change point model (MVCM) to detect CNVs or breakpoints with an approximate *p*-value for statistical testing. In addition, Oldridge et al. [14] quantitatively evaluated several processing and segmentation strategies when short-sequence oligonucleotide arrays are applied and provide guidelines to optimize performance based on study-specific objectives.

Besides the above mentioned CNV detection methods, high-density single nucleotide polymorphism (SNP) arrays are becoming more and more popular in CNV detection. The main reason is that the inheritance pattern and linkage disequilibrium of CNVs are similar to those of the SNPs [6,7]. Zhao et al. [15] measured the locus-specific hybridization intensity by hybridizing genomic representations of both DNA to SNP arrays to detect some novel CNVs in cancer samples. Huang et al. [16] proposed an algorithm that used whole genome sampling analysis (WGSA) by jointly measuring perfect match intensity and discrimination ratios to identify copy number changes. An QuantiSNP algorithm [17] described in 2007 used Objective Bayes Hidden-Markov Model (OB-HMM) by incorporating log R ratio and B allele frequency to set certain hyperparameters as priors, and using a novel re-sampling framework to calibrate the model to a fixed false positive error rate for CNVs detection. Wang et al. [18] designed a tool named PennCNV, a hidden Markov model (HMM) based approach, for kilobase-resolution detection of CNVs by combining multiple sources of information together, not only the total signal intensity and allelic intensity ratio of each SNP marker, but also the neighboring distances, the allele frequency and the pedigree information.

Recently, disease classification using copy number variation data has been demonstrated by several groups [19-22]. The motivation of this kind of method is that appropriate machine learning models for disease classification using copy number variation data will be effective not only for clinical treatment, but also for identification of disease susceptibility loci. Generally, copy numbers at probe loci are used directly as features. In a CNV data set, the association between a disease and a set of relevant CNVs are investigated. Patients and normals are often categorized in groups according to their copy number changes. Thousands of CNVs in different regions of chromosomes are used to describe characteristics of patient/normal samples.

When many CNVs are used to detect the association between a disease and multiple marker genotypes, we expect in a typical data set that contains the CNV data of several thousands of CNVs in different individuals, it is common to find only several numbers of copy number positions having genetic patterns that are highly specific to each group of individuals. The CNVs are called the relevant CNVs, as opposed to the irrelevant CNVs that do not help much in identifying the group (i.e., individuals of the same type). Due to the large number of CNVs being irrelevant to each group, two individuals in the same group could have low similarity when measured by a simple similarity function that consider the characteristics of all CNVs. The groups may thus be undetectable by classification algorithms. Here, we are interested in the development of high-dimensional numerical classification algorithm that can identify group of individuals and their relevant CNVs, i.e., detect the association between a disease and multiple marker variations. In this paper, we address this problem by applying a shrunken dissimilarity measure to copy number variation values derived from genome-wide SNP genotyping data. Performance was measured via cross-validation classification accuracy. By shrinkage, the number of relevant CNVs can be determined. In order to understand relevant CNVs, we study their corresponding SNPs in the genome and a statistical software PLINK is employed to compute the pair-wise SNP-SNP interactions, and identify SNP network based on their *P*-values. Some statistical analysis are done to illustrate the significance of our selected SNP networks. For the unique genes that those SNPs are located in, a gene-gene similarity value is computed using GOSemSim and gene pairs that has a similarity value being greater than a threshold are selected to construct gene networks. A gene enrichment analysis are done to show that some molecular functions and biological process are significantly associated with our gene networks. The whole framework indicates that our classification model is efficient in high dimensional CNV detection and SNP and gene networks constructed afterwards have relationships directly or indirectly to disease study.

The outline of this paper is given as follows. In Section 2, we propose the LRR calculation for signal intensities, the shrunken dissimilarity measure to analyze CNV data classification and the logistic model to calculate SNP interactions. In Section 3, we present experimental results on two real CNV data sets derived from genome-wide SNP arrays, and illustrate their corresponding SNP and gene networks for statistical and biological analysis. Finally, we give concluding remarks in Section 4.

## Methods

### LRR calculation

There are many available tools that can be used to do genotyping call and Log R Ratio (LRR) calculation, such as Birdsuite [23], CNAG [24], dChip [25,26] and GLAD [27]. We calculated LRR values using PennCNV [18], which is a public available software for copy number variation detection from SNP genotyping arrays. For each SNP in a genome-wide data, the raw signal intensity values for its two alleles A and B are measured and then subject to a normalization procedure. The normalized signal intensity values should reveal three clusters (AA, AB, BB) representing three distinct genotypes. A genotype calling is a genotype assignment based on these three canonical clusters.

The normalization procedure produces *X* and *Y* values for each SNP, representing the experiment-wide normalized signal intensity on alleles A and B, respectively. One additional measurement will then be calculated for each SNP, where *R* = *X* + *Y* refers to the total signal intensity. As for a normalized measure of total signal intensity, the LRR value for each SNP is then calculated as

*LRR* = *log*_2_(*R_observed_*/*R_expected_*) (1)

where *R_expected_* is computed from linear interpolation of canonical genotype clusters [28]. LRR refers to the logarithm (in base 2) of the total observed normalized intensity *R_observed_* of the two alleles *A* and *B* relative to the expected *R_expected_*. For normal diploid genotypes, LRR should fluctuate randomly around zero.

### Shrunken centroid method

It is well-known that DNA microarray data, which can simultaneously measure the expression level of thousands of different genes, have been successfully used to identify genetic heterogeneity of disease. However, mircoarray data typically has a large number of genes (features) and relatively few samples (observations), meaning that conventional machine learning methods may fail when applied to such data. The Prediction Analysis for Microarrays (PAM), has recently been reported as a potential powerful tool for microarrays analysis, which is based on the technique of nearest shrunken centroid [29,30]. Nearest shrunken centroid is the “de-noised” version of simple nearest centroid classification. The main idea of nearest centroid classification is to compare test sample to each class centroid. The class whose centroid the test sample is closest to is the predicted class for test sample. The centroid is the average value for each feature (or attribute) in each class, normalized by the pooled within-class standard deviation. The nearest shrinkage centroid classification shrinks each centroid toward the overall centroid for all classes by a certain amount, which is called a “threshold”. After the overall centroid is set to zero, this shrinkage is to move the centroid to zero by the threshold, i.e., if the value of centroid in magnitude is larger than the threshold, it is subtracted by the threshold; if it is less than the threshold, it is set to zero, so the corresponding feature disappears, and it isn’t used for the following classification. By doing so, nearest shrunken centroid can find out the minimal subset of genes which succinctly characterize each class. Shrunken centroid method is traditionally used to deal with microarray data. In this paper, we adopt this method for copy number variation values derived from SNP genotyping arrays. The normalized signal intensity Log R Ratio (LRR) value in each probe loci calculated from existing software, which we will give a detailed description in the next subsection, will be considered as a unique feature.

Suppose there are *n* samples, *p* CNVs, and *K* classes. For *i*th CNV, the class centroid  is the average value of LRR values within one class, and the overall centroid  is the average value over all classes. The difference between class centroid and overall centroid is normalized by pooled with-class standard deviation as follows:(2)

where

*C_k_* denote the indices of the *n_k_* samples in class *k*, and *s*_0_ is a positive constant included to prevent the possibility that a CNV with a low LRR level could produce a large *d_ij_*. Equation (2) can be rewritten as:(3)

Then the shrunken centroid is defined as:(4)

where , and *t*_+_ = *t* if *t* > 0 and zero otherwise. The parameter Δ is usually sought by cross-validation with minimal classification error rate. Note that if  for all *k* for a given *i*, then all of the shrunken centroids are zero, and the *i*th CNV does not contribute to the classification process. For prediction, the class label of test sample **t** is determined by the nearest shrunken centroid:

where

*π_k_* is the prior probability of class k.

### Logistic model to calculate SNP interactions

As our experimental data sets are both disease-trait samples, it is feasible to test epistasis using PLINK to [31] detect SNP-SNP interactions, which is a free, open-source whole genome association analysis toolset, designed to perform a range of basic, large-scale analysis in a computationally efficient manner. All pairwise combinations of input SNPs can be tested using a logistic regression model, which is based on allele dosage for each SNP, A and B, and fits the model in the form of (5)

*Y*~*b*_0_ + *b*_1_*.A* + *b*_2_*.B* + *b*_3_*.AB* + *e* (5)

The test for interaction is based on the coefficient of *b*_3_, therefore only considers allelic by allelic epistasis. We focus on symmetrical cases in our study, that means only unique pairs are analysed, for example, if SNP1*SNP2 is performed, SNP2*SNP1 will not be calculated again. The *χ*^2^ statistics is applied and the odds ratio for interaction is interpreted in the standard manner: a value of 1.0 indicates no effect.

## Results and Discussion

### WTCCC Type 1 Diabetes study

#### Data set and preprocessing

The core study of the Wellcome Trust Case Control Consortium (WTCCC) comprised an analysis of genetic signals from each of seven common human diseases (type 1 diabetes, type 2 diabetes, coronary artery disease, hypertension, bipolar disorder, rheumatoid arthritis and Croh disease) [32]. Genotyping for type 1 diabetes (T1D) study was conducted by Affymetrix using the (“commercial”) Affymetrix 500K chip in 3504 samples with 2000 cases and 1504 controls.

The quantile normalized signal intensity data, which were generated from the Affymetrix intensity (‘CEL’) files and used as input to the Chiamo genotype calling program, was downloaded from WTCCC webpage and appropriately prepared according to the requirements of input file formats of PennCNV. A biological literature searching indicates that in chromosome 6, there are some well-known T1D related genes. Therefore, we chose chromosome 6 as an example to demonstrate our method. There are 31470 unique SNP loci in this chromosome, 1 markers have complete genotyping failure with confidence threshold of 0*.*01, 1042 markers do not have at least two types of genotypes, 2460 markers have abnormal patterns, so there are 27967 SNP markers have been analyzed to construct canonical clustering positions.

#### Classification results

**Figure 1:**
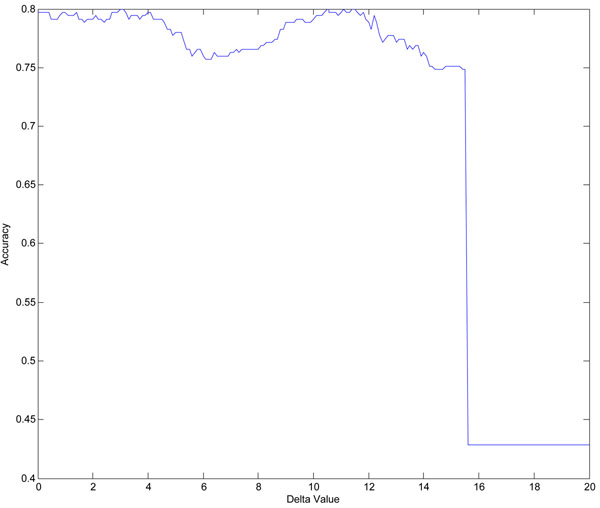
**Relationship between Δ and accuracy in chromosome 6 of T1D study.** Illustration of the accuracy obtained in chromosome 6 of Type 1 diabetes data set when change Δ value from 0 to 20. For chromosome 6, in each trial, all the 3504 samples of both control and case were randomly divided into 10 equal partitions. For each of the 10 partition groups, we selected one of them as testing set and the remaining nine of them were considered as training sets. 10 trials were considered and the results were collected based on this 10-fold cross validation procedure. This figure was drawn based on one of these ten trails when the highest accuracy (accuracy refers to the percentage of correctly classified samples over all test samples) was obtained. X axis refers to Δ value, it increases from 0 to 20. Y axis refers to the accuracy obtained in chromosome 6 when using our method, it fluctuates when different Δ values are applied and the highest accuracy is obtained when Δ is equal to 10.56.

After a 10-fold cross validation setting, 3154 samples were selected as training data and the remaining 350 samples would be the testing data. The highest classification accuracy (correctly classified samples in testing data sets in the 10-fold cross validation) in the 10 trials is 80%. The parameter Δ was tuned to obtain the highest accuracy in the test. Figure 1 shows the relationships between Δ values and accuracies obtained in this trial. We can observe that when the value of Δ is increased from 0 to 20, the classification accuracy fluctuates and achieves the highest one of 80*.*00% when Δ = 10*.*56.

Our method can select 63 SNPs, 25 of them are located in gene coding areas. In order to investigate how this small set of SNPs can distinguish case and control people so well, we further discovered the distribution of each SNP in case and control group. Table 1 shows the average and variance of LRR values in each SNP position within case and control group respectively. We only considered the SNPs that are located in gene coding areas here. We gave a ranking for these SNPs based on the parameter of  mentioned in the previous section, which can be considered as the contribution of this SNP to the classification problem. We can see that in each SNP position, the distribution of LRR values within case people and control people are significantly different, which are consistent with our classification results.

**Table 1:** Distribution of LRR values for selected SNPs of T1D study.

SNP ID		Control		Case	
		mean	variance	mean	variance
rs6940177	2.3940	0.0522	0.0431	-0.1845	0.0402
rs17209874	2.2398	0.1071	0.0288	-0.0926	0.0218
rs9479373	2.1075	0.1027	0.0187	-0.0838	0.0213
rs3778077	1.8133	0.0937	0.0157	-0.1180	0.0450
rs16875181	1.6937	0.1107	0.0216	-0.1033	0.0454
rs7754428	1.6853	0.0968	0.0199	-0.0921	0.0262
rs6902158	1.6838	0.0888	0.0278	-0.1067	0.0244
rs6912853	1.3226	0.1116	0.0254	-0.0770	0.0274
rs991974	1.2833	0.0912	0.0177	-0.1010	0.0351
rs643394	1.2636	0.1146	0.0244	-0.0943	0.0471
rs9397339	1.1925	0.0906	0.0177	-0.0906	0.0264
rs319123	1.1473	0.1015	0.0199	-0.0863	0.0309
rs352095	0.9764	0.1097	0.0380	-0.0873	0.0310
rs1738262	0.7388	0.0979	0.0249	-0.0860	0.0311
rs7760230	0.5918	0.0914	0.0171	-0.0730	0.0204
rs10947885	0.4503	0.0663	0.0166	-0.1074	0.0308
rs1334689	0.4209	0.1052	0.0197	-0.0775	0.0361
rs1406882	0.3947	0.1047	0.0236	-0.0837	0.0402
rs1563666	0.2513	0.0916	0.0329	-0.0846	0.0245
rs9321142	0.2245	0.0843	0.0140	-0.0719	0.0210
rs2073012	0.1519	0.0989	0.0281	-0.0668	0.0208
rs11154452	0.1286	0.0847	0.0243	-0.0905	0.0324
rs9394755	0.1208	0.0980	0.0270	-0.0714	0.0257
rs9371491	0.0910	0.0832	0.0151	-0.0732	0.0217
rs6918886	0.0069	0.0951	0.0220	-0.0922	0.0473

#### SNP network construction and analysis

All the SNPs selected by our method can be divided into two categories, those are located in gene coding areas and those are not. We did a statistical analysis between these two categories of SNPs using PLINK [31]. All pairwise combinations of SNPs can be tested. Odds ratio for interaction, *χ*^2^ statistic and asymptotic *P*-value will be provided in the output file. When adopted different thresholds, different kinds of SNP networks can be constructed. Here “threshold” means only SNP pairs that have a P-value smaller than this threshold will be considered and be included in the SNP network. By constructing these SNP networks, we can figure out some potential SNP-SNP interactions that are still unknown. Table 2 shows a detailed characteristics of SNP networks under different thresholds, including the number of SNP networks, the number of nodes and edges in each individual network. As we were interested at SNP-SNP interactions, and we only considered the networks where the number of SNPs are more than one. We can see that the number of networks increases when the significant P-value decreases as one single network can be separated into several smaller ones. However, when P-value further decreases, the number of networks is reduced until there is only one left as there are too small SNPs to form a network.

**Table 2:** Characteristics of SNP networks in different thresholds of T1D study.

PLINK thresholds	NO. of networks	NO. of SNPs	NO. of SNP pairs
0.1	2	1(2), 2(49)	1(1), 2(60)
0.05	4	1(3), 2(4), 3(9), 4(20)	1(2), 2(3), 3(8), 4(21)
0.01	5	1(2), 2(2), 3(2), 4(2), 5(5)	1(1), 2(1), 3(1), 4(1), 5(4)
0.005	3	1(2), 2(2), 3(5)	1(1), 2(1), 3(4)
0.001	1	1(2)	1(1)

**Figure 2:**
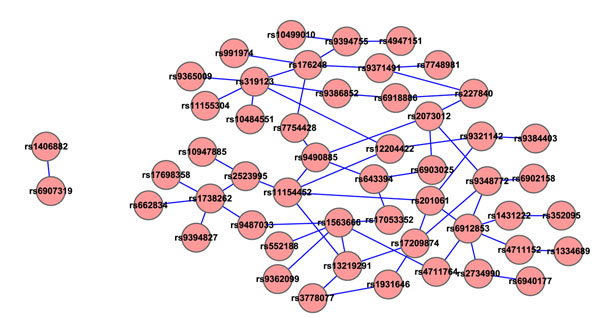
**SNP networks when PLINK threshold=0.1 of T1D study.** SNP networks were constructed based on the *P*-value of PLINK epistasis test using Cytoscape, all the pairwise SNP-SNP interactions that had a PLINK *P*-value smaller than 0.1 were involved in this SNP network. Each node in the figure is labeled as its SNP ID and the edge between two SNPs indicates whether this pair of SNPs are interacted under a *P* < 0*.*1 significance level. This network includes 2 individual networks and 51 SNPs.

**Figure 3:**
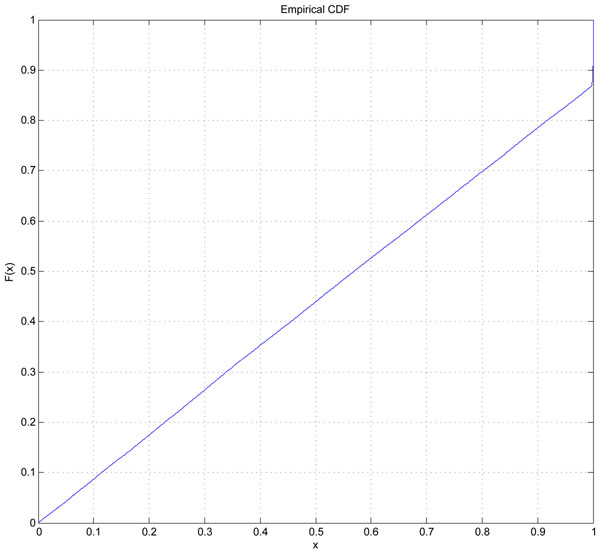
**Cumulative distribution of 500 random SNP networks.** Cumulative distribution of 500 random SNP networks. Each of these 500 random networks maintained the same size with our selected SNP network (49 SNPs). SNPs were selected randomly, calculated all pairwise SNP-SNP interactions using PLINK, got the average P-value of all pairs. This cumulative distribution is a statistics of these 500 random networks with X axis is the average P-value within this network and Y axis is the cumulative probability that within a particular X threshold.

To further utilize and benefit from these SNP networks selected by our method, we next performed some statistical analysis. For each network under one particular threshold, we randomly picked the same number of SNPs with our selected SNP network to get a random SNP network, calculated all the pairwise SNP-SNP interactions and got the average value of all the P-values within this random network. Such process were repeated 500 times. Table 3 shows the significance of our selected networks compared with random ones. As SNP networks under threshold of 0.1 almost involve all the SNPs selected by our shrunken classification model, we chose this threshold as an example to further analyze. Figure 2 shows the two SNP networks under this threshold with one is smaller, including only 2 SNPs, but the other one is much bigger, including 49 unique SNPs. Figure 3 is the cumulative distribution of the average P-values for all 500 samples (each with 49 SNPs). Compared with the distribution of random samples, which was with mean of 0.5645 and variance of 0.1008, our selected SNP network (average P-value is 0.0450) is very significant, with significance value of 1*.*2765 × 10^–7^.

**Table 3:** Significance of selected SNP networks in different thresholds of T1D study.

PLINK thresholds	NO. of networks	Selected mean	Random		Significance value
			mean	variance	
0.1	2	1(0.0985)	1(0.5648)	1(0.0991)	1.2672 × 10–6
		2(0.0450)	2(0.5645)	2(0.1008)	1.2765 × 10–7
0.05	4	1(0.0298)	1(0.5805)	1(0.1018)	3.1578 × 10–8
		2(0.0225)	2(0.5682)	2(0.0995)	2.0741 × 10–8
		3(0.0306)	3(0.5638)	3(0.1005)	5.6191 × 10–8
		4(0.0203)	4(0.5633)	4(0.1002)	2.9939 × 10–8
0.01	5	1(0.0054)	1(0.5862)	1(0.1003)	3.5062 × 10–9
		2(0.0057)	2(0.5645)	2(0.0983)	6.5545 × 10–9
		3(0.0012)	3(0.5622)	3(0.0971)	3.7898 × 10–9
		4(0.0011)	4(0.5545)	4(0.1009)	2.0716 × 10–8
		5(0.0015)	5(0.5528)	5(0.1004)	1.9979 × 10–8
0.005	3	1(0.0011)	1(0.5327)	1(0.1024)	1.0436 × 10–7
		2(0.0012)	2(0.5300)	2(0.0998)	5.8348 × 10–8
		3(0.0015)	3(0.5762)	3(0.1004)	5.1991 × 10–9
0.001	1	1(0.0001)	1(0.5578)	1(0.0950)	2.1721 × 10–9

#### Gene network construction and analysis

Our method can select 63 SNPs, 25 of them are located in gene coding areas, and these 25 SNPs belong to 24 unique genes, which are all shown in Table 4. After checking with NCBI, we found that some of the genes that our selected SNPs located in are directly or indirectly related to diabetes. For example, GMDS refers to GDP-mannose 4,6-dehydratase, which catalyzes the conversion of GDP-mannose to GDP-4-keto-6-deoxymannose, and it has been verified that mannose-binding lectin is a predictor of Type 1 Diabetes [33]. MTHFD1L is an enzyme involved in THF synthesis in mitochondria, and mutations of mitochondria strongly associate with diabetes [34]. And also for LAMA2, whose full name is laminin, alpha 2, is a major component of the basement membrane, the abnormal level of it has been reported to have significant relationship to the presence of diabetes [35].

We computed all the pair-wise functional similarities of these 24 gene products using GOSemSim, which is an open source and open development software project for the analysis and comprehension of genomic data running in the platform of R Bioconductor package [36]. GOSemSim estimates the similarity scores of gene pairs according to their Gene Ontology (GO) [37] terms: molecular function (MF), biological process (BP) and cellular component (CC). In this paper, we only considered two of these terms: MF and BP and adopted Rel’s method [38] to compute the similarity values, which is based on the information content of the GO terms and define information content as the frequency of each term occurs in the GO corpus. Afterwards, gene pairs that have a similarity value being greater than a threshold, were selected to construct a gene network using Cytoscape [39]. As we were interested at gene-gene interactions, and we only considered the networks where the number of genes are more than one. In Table 5, we showed the number of gene networks formed by using different threshold values and the number of pairs of genes involved.

**Table 4:** Detailed gene descriptions of SNPs selected in chromosome 6 of T1D study.

SNP ID	Gene Symbol	Gene ID	Description
rs6940177	TRDN	10345	triadin
rs17209874	RUNX2	860	runt-related transcription factor 2
rs9479373	LOC646024	646024	locus-region
rs3778077	NUDT3	11165	nudix (nucleoside diphosphate linked moiety X)-type motif 3
rs16875181	GPR116	221395	G protein-coupled receptor 116
rs7754428	GMDS	2762	GDP-mannose 4,6-dehydratase
rs6902158	LRRC16	55604	leucine rich repeat containing 16
rs6912853	BTN3A1	11119	butyrophilin, subfamily 3, member A1
rs991974	LMBRD1	55788	chromosome 6 open reading frame 209
rs643394	LOC442256	442256	similar to PPP1R14B protein
rs9397339	PLEKHG1	57480	pleckstrin homology domain containing, family G (with RhoGef domain) member 1
rs319123	C6orf210	57107	chromosome 6 open reading frame 210
rs352095	FLJ34503	285759	hypothetical protein FLJ34503
rs1738262	DNAH8	1769	dynein, axonemal, heavy polypeptide 8
rs7760230	SNX9	51429	sorting nexin 9
rs10947885	LRFN2	57497	leucine rich repeat and fibronectin type III domain containing 2
rs1334689	EPB41L2	2037	erythrocyte membrane protein band 4.1-like 2
rs1406882	FYN	2534	FYN oncogene related to SRC, FGR, YES
rs1563666	LOC643281	643281	intron
rs9321142	LAMA2	3908	laminin, alpha 2 (merosin, congenital muscular dystrophy)
rs2073012	NOX3	50508	NADPH oxidase 3
rs11154452	LAMA2	3908	laminin, alpha 2 (merosin, congenital muscular dystrophy)
rs9394755	UNC5CL	222643	unc-5 homolog C (C. elegans)-like
rs9371491	MTHFD1L	25902	methylenetetrahydrofolate dehydrogenase (NADP+ dependent) 1-like an enzyme involved in THF synthesis in mitochondria
rs6918886	RPS6KA2	6196	ribosomal protein S6 kinase, 90kDa, polypeptide 2

**Table 5:** Gene networks formed for different threshold values of T1D study.

GOSemSim thresholds	Number of gene pairs	Number of gene networks
0.20	1	1
0.19	11	2
0.15-0.18	12	3
0.14	14	2
0.13	24	1

**Figure 4:**
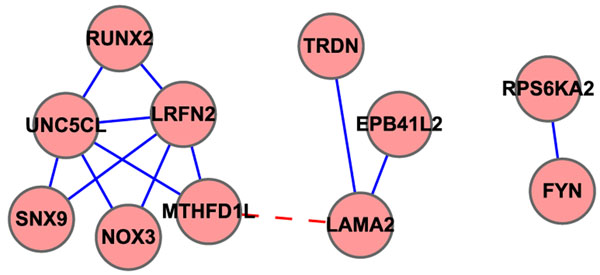
**Gene network when GOSemSim threshold=0.15 of T1D study.** Gene network constructed using Cytoscape. In chromosome 6 of T1D data, all SNPs selected belong to 24 unique genes. All the pairwise similarity values of these 24 genes were computed using GOSemSim and gene pairs that had a > 0*.*15 threshold were grouped together. Every node in the figure is labeled as its gene symbol and the edge between two genes indicates whether this pair of genes has a > 0*.*15 threshold or not. This network includes 3 individual networks and totally 11 genes.

We can see in Table 5 that the number of gene networks increases when the threshold value increases as more networks are formed. However, when threshold value further increases, the number of networks is reduced until there is only one network left. According to Table 5, we selected the threshold of 0.15 for analysis as the number of gene networks is highest than those using other threshold values. Figure 4 demonstrates the gene networks constructed by our method when threshold is equal to 0.15. Gene pairs that are grouped in the same network suggested a strong potential for interaction effects in biological process. We can see from this figure that there are 3 networks, including 12 pairs and 11 unique genes.

We found some interesting relationships from SNP and gene networks. For example, for SNPs rs9321142 and rs9371491, which are interacted in the same SNP network under *P*-value of 0.1 in Figure 2, their corresponding genes are LAMA2 and MTHFD1L, but located in different gene networks in Figure 4, which means that maybe we can merge these two gene networks together, (shown with red dash line in Figure 4), and furthermore, can be consistent with existing biological functions of these two genes to diabetes. Another example, rs6912853 is interacted with rs2734990 under a very significant P-value of 9*.*43 x 10*^–^*^5^, but rs2734990 is located in intergenic area and do not have a record in Gene Ontology until now, maybe we can make use of rs6912853’s gene information, BTN3A1, to further analyze the inner functions of rs2734990 and extend GO afterward.

**Figure 5:**
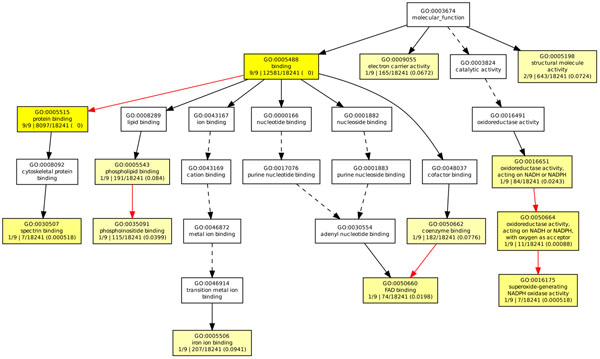
**GOEAST analysis in molecular function category of T1D study.** The GOEAST graphical output of enriched GO terms in the molecular function category for our selected gene network of T1D study. Boxes represent GO terms, labeled by its GO ID, term definition, and detailed information, organized as “*q*/*m*|*t*/*k*(*p* – *value*)”, where *q* is the count of genes associated with the listed GOID (directly or indirectly) in our dataset, *m* is the count of genes associated with the listed GOID (directly or indirectly) on the chosen platform, *k* is the total number of genes in our dataset, *t* is the total number of genes on the chosen platform, *p*-value is of the significance for the enrichment in the dataset of the listed GOID under hypergeometric distribution. Significantly enriched GO terms are marked yellow. The degree of color saturation of each node is positively correlated with the enrichment significance of the corresponding GO term. Nonsignificant GO terms within the hierarchical tree are shown as white boxes. Branches of the GO hierarchical tree without significantly enriched GO terms are not shown. Arrows represent connections between different GO terms. Red arrows represent relationships between two enriched GO terms, black solid arrows represent relationships between enriched and unenriched terms and black dashed arrows represent relationships between two unenriched GO terms.

**Figure 6:**
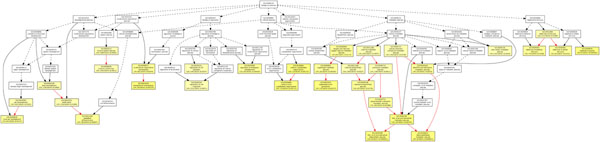
**GOEAST analysis in biological process category of T1D study.** The GOEAST graphical output of enriched GO terms in the biological process category for our selected gene network of T1D study. Boxes represent GO terms, labeled by its GO ID, term definition, and detailed information, organized as “*q*/*m*|*t*/*k*(*p* – *value*)”, where *q* is the count of genes associated with the listed GOID (directly or indirectly) in our dataset, *m* is the count of genes associated with the listed GOID (directly or indirectly) on the chosen platform, *k* is the total number of genes in our dataset, *t* is the total number of genes on the chosen platform, *p*-value is of the significance for the enrichment in the dataset of the listed GOID under hypergeometric distribution. Significantly enriched GO terms are marked yellow. The degree of color saturation of each node is positively correlated with the enrichment significance of the corresponding GO term. Nonsignificant GO terms within the hierarchical tree are shown as white boxes. Branches of the GO hierarchical tree without significantly enriched GO terms are not shown. Arrows represent connections between different GO terms. Red arrows represent relationships between two enriched GO terms, black solid arrows represent relationships between enriched and unenriched terms and black dashed arrows represent relationships between two unenriched GO terms.

**Figure 7:**
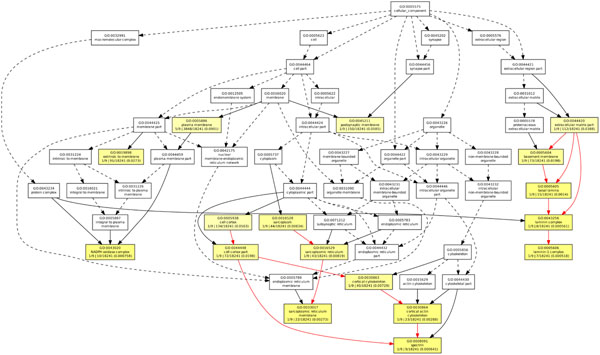
**GOEAST analysis in cellular component category of T1D study.** The GOEAST graphical output of enriched GO terms in the cellular component category for our selected gene network of T1D study. Boxes represent GO terms, labeled by its GO ID, term definition, and detailed information, organized as “*q*/*m*|*t*/*k*(*p* – *value*)”, where *q* is the count of genes associated with the listed GOID (directly or indirectly) in our dataset, *m* is the count of genes associated with the listed GOID (directly or indirectly) on the chosen platform, *k* is the total number of genes in our dataset, *t* is the total number of genes on the chosen platform, *p*-value is of the significance for the enrichment in the dataset of the listed GOID under hypergeometric distribution. Significantly enriched GO terms are marked yellow. The degree of color saturation of each node is positively correlated with the enrichment significance of the corresponding GO term. Nonsignificant GO terms within the hierarchical tree are shown as white boxes. Branches of the GO hierarchical tree without significantly enriched GO terms are not shown. Arrows represent connections between different GO terms. Red arrows represent relationships between two enriched GO terms, black solid arrows represent relationships between enriched and unenriched terms and black dashed arrows represent relationships between two unenriched GO terms.

In order to investigate the inner functions of the genes involved in our gene network in an ontology level, we performed the GO enrichment analysis by GOEAST [40], which is a web-based software toolkit for fast identification of underlining biological relevance of high-throughput experimental results. GOEAST discovers statistically significantly enriched GO terms among the given gene list based on their hypergeometric probability. So the purpose of this analysis here is to find out which GO terms can be strongly enriched or significantly associated with our selected genes. We did a Batch-Genes search using “Homo sapiens” background for all the three GO categories: “molecular function”, “biological process” and “cellular component”, as shown in Figure 5, Figure 6 and Figure 7 respectively. In these figures, boxes represent GO terms, labeled by its GOID, term definition, *P*-value and detail information. Significantly enriched GO terms are marked yellow. The degree of color saturation of each node is positively correlated with the significance of enrichment of the corresponding GO term. The detail information labeled in the enriched GO nodes are organized as “*q*/*m*\*t*/*k*(*p* – *value*)”, where *q* is the count of genes associated with the listed GOID (directly or indirectly) in our dataset, *m* is the count of genes associated with the listed GOID (directly or indirectly) on the chosen platform, *k* is the total number of genes in our dataset, *t* is the total number of genes on the chosen platform, *p*-value is of the significance for the enrichment in the dataset of the listed GOID under hypergeometric distribution. We also extracted all the GO terms and the corresponding involved genes with a significant *P* < 0*.*001, see Table 6. We highlighted the GO terms that may be related to diabetes, which need to be further verified and investigated by biologists.

**Table 6:** Gene Ontology terms significant associated with selected genes (*P* < 0.001) of T1D study.

GO Category	GO ID	GO Annotation	Genes	*P*-value
**Molecular Function**	**GO:0016175**	**superoxide-generating NADPH oxidase activity**	**NOX3**	**0.000518**
Molecular Function	GO:0030507	spectrin binding	EPB41L2	0.000518
Cellular Component	GO:0005606	laminin-1 complex	LAMA2	0.000518
Biological Process	GO:0009629	response to gravity	NOX3	0.000561
**Biological Process**	**GO:0009396**	**folic acid and derivative biosynthetic process**	**MTHFD1L**	**0.000561**
Cellular Component	GO:0043256	laminin complex	LAMA2	0.000561
**Biological Process**	**GO:0046653**	**tetrahydrofolate metabolic process**	**MTHFD1L**	**0.000641**
Cellular Component	GO:0008091	spectrin	EPB41L2	0.000641
**Cellular Component**	**GO:0043020**	**NADPH oxidase complex**	**NOX3**	**0.000758**
**Molecular Function**	**GO:0050664**	**oxidoreductase activity, acting on NADH or NADPH, with oxygen as acceptor**	**NOX3**	**0.000880**

### Hirschsprung study

#### Data set and preprocessing

Hirschsprung (HSCR, MIM 142623), also known as aganglionic megacolon, is a congenital intestinal disease. Patients suffer from different extent of aganglionosis due to the absence of ganglion cells in the gastrointestinal tract. Trisomy 21 (Down syndrome) is the most recurrent structural variations found in HSCR, contributing to 2-10% of the cases.

This data set consists of 330 controls and 121 cases of Chinese ethnicity. In this analysis, we excluded the centromeric and telomeric regions using 500kb threshold because these regions tend to have bias in CNV calling. We transformed the probe intensities into LRR values from this SNP data set (n=3369) using PennCNV with the same parameter setting and procedure as mentioned above.

#### Classification results

**Figure 8:**
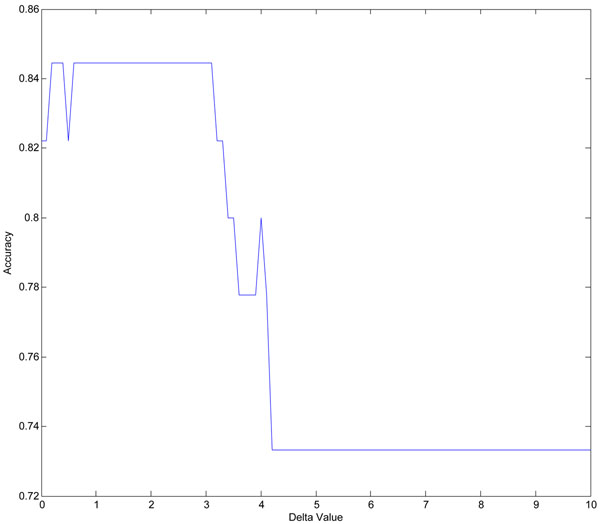
**Relationship between Delta and accuracy in chromosome 21 of Hirschsprung study.** Illustration of the accuracy obtained in chromosome 21 of Hirschsprung data set when change Δ value from 0 to 10. For chromosome 21, in each trial, all the 451 samples of both control and case were randomly divided into 10 equal partitions. For each of the 10 partition groups, we selected one of them as testing set and the remaining nine of them were considered as training sets. 10 trials were considered and the results were collected based on this 10-fold cross validation procedure. This figure was drawn based on one of these ten trails when the highest accuracy (accuracy refers to the percentage of correctly classified samples over all test samples) was obtained. X axis refers to Δ value, it increases from 0 to 10. Y axis refers to the accuracy obtained in chromosome 21 when using our method, it fluctuates when different Δ values are applied and the highest accuracy is obtained when Δ is equal to 3.1.

After a 10-fold cross validation, 406 samples were selected as the training set and the remaining 45 samples were used as the testing set. Figure 8 illustrated the relationship of Δ and accuracy for our method. Our method achieved the optimal accuracy of 84*.*44%, when the threshold of Δ was set either between 0*.*2 – 0*.*4 or 0*.*6 – 3*.*1. We showed all the 6 SNPs selected when Δ is equal to 3*.*1 in Table 7, it includes the average and variance of LRR values in each SNP position within case and control group respectively.

**Table 7:** Distribution of LRR values for selected SNPs of Hirschsprung study.

SNP ID		Control		Case	
		mean	variance	mean	variance
rs11701130	1.0256	-0.1325	0.1130	0.1821	0.0686
rs2837770	0.4607	-0.1387	0.1014	0.1208	0.0578
rs2824050	0.3089	-0.0559	0.0331	0.1185	0.0214
rs928862	0.2301	-0.0934	0.0632	0.1193	0.0339
rs2824724	0.2177	-0.1012	0.1169	0.1439	0.0626
rs845930	0.1205	-0.1543	0.2580	0.1668	0.1373

As there are only 6 SNPs selected, the number is too small to construct any networks. So for this dataset, we did not do any analysis in SNP level.

#### Gene network construction and analysis

**Figure 9:**
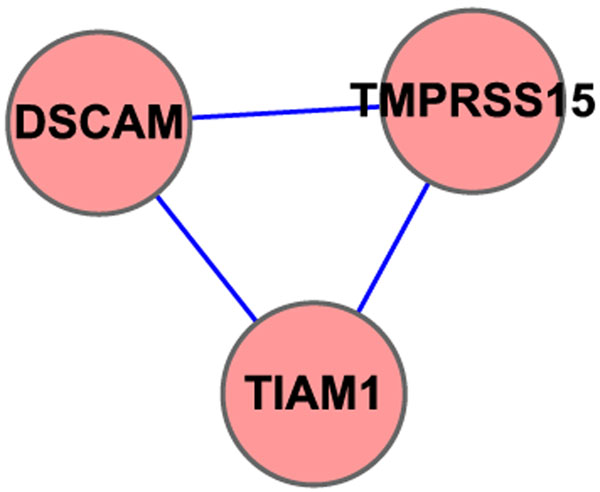
**Gene network when GOSemSim threshold=0.01 of Hirschsprung study.** Gene network constructed using Cytoscape. In chromosome 21 of Hirschsprung data, all SNPs selected belong to 3 unique genes. All the pairwise similarity values of these 3 genes were computed using GOSemSim and gene pairs that had a > 0*.*01 threshold were grouped together. Every node in the figure is labeled as its gene symbol and the edge between two genes indicates whether this pair of genes has a > 0*.*01 threshold or not. This network includes 1 individual network and 3 unique genes.

All the 6 SNPs selected by our method belong to 3 unique genes. The detailed gene information can be seen in Table 8. We calculated all the pairwise gene similarity values with the same parameter settings of previous data set. If define a threshold of 0.01, all these 3 genes can be linked together, see Figure 9. After a survey in the literature about the known findings of these 3 genes, we found some interesting phenomena. For gene DSCAM, which is a previously implicated gene for the involvement of developing hirschsprung disease [41,42], and for gene TMPRSS15, whose mutations can cause enterokinase deficiency, a malabsorption disorder characterized by diarrhea and failure to thrive, both of these two genes have a biological record that directly to hirschsprung disease, and both of them can be selected as relevant features using our method, furthermore, can be grouped together in our gene network, which can strongly prove the efficiency of our model.

**Table 8:** Detailed descriptions of SNPs selected in chromosome 21 of Hirschsprung study.

SNP ID	Gene Symbol	Gene ID	Description
rs11701130	intergenic		
rs2837770	DSCAM	1826	Down syndrome cell adhesion molecule
rs2824050	intergenic		
rs928862	intergenic		
rs2824724	TMPRSS15	5651	Mutations in this gene cause enterokinase deficiency, a malabsorption disorder characterized by diarrhea and failure to thrive
rs845930	TIAM1	7074	T-cell lymphoma invasion and metastasis 1

**Figure 10:**
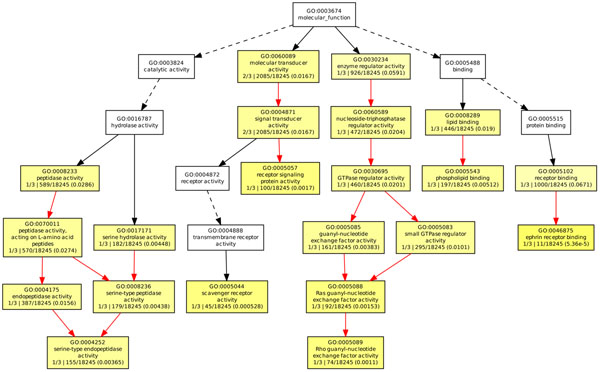
**GOEAST analysis in molecular function category of Hirschsprung study.** The GOEAST graphical output of enriched GO terms in the molecular function category for our selected gene network of Hirschsprung study. Boxes represent GO terms, labeled by its GO ID, term definition, and detailed information, organized as “*q*/*m*|*t/k*(*p* – *value*)”, where *q* is the count of genes associated with the listed GOID (directly or indirectly) in our dataset, *m* is the count of genes associated with the listed GOID (directly or indirectly) on the chosen platform, *k* is the total number of genes in our dataset, *t* is the total number of genes on the chosen platform, *p*–value is of the significance for the enrichment in the dataset of the listed GOID under hypergeometric distribution. Significantly enriched GO terms are marked yellow. The degree of color saturation of each node is positively correlated with the enrichment significance of the corresponding GO term. Nonsignificant GO terms within the hierarchical tree are shown as white boxes. Branches of the GO hierarchical tree without significantly enriched GO terms are not shown. Arrows represent connections between different GO terms. Red arrows represent relationships between two enriched GO terms, black solid arrows represent relationships between enriched and unenriched terms and black dashed arrows represent relationships between two unenriched GO terms.

**Figure 11:**
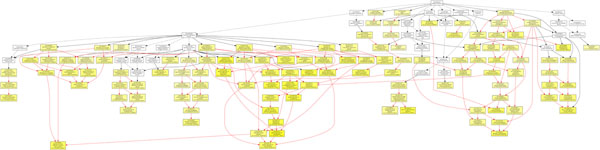
**GOEAST analysis in biological process category of Hirschsprung study.** The GOEAST graphical output of enriched GO terms in the biological process category for our selected gene network of Hirschsprung study. Boxes represent GO terms, labeled by its GO ID, term definition, and detailed information, organized as “*q*/*m*|*t*/*k*(*p* – *value*)”, where *q* is the count of genes associated with the listed GOID (directly or indirectly) in our dataset, *m* is the count of genes associated with the listed GOID (directly or indirectly) on the chosen platform, *k* is the total number of genes in our dataset, *t* is the total number of genes on the chosen platform, *p*-value is of the significance for the enrichment in the dataset of the listed GOID under hypergeometric distribution. Significantly enriched GO terms are marked yellow. The degree of color saturation of each node is positively correlated with the enrichment significance of the corresponding GO term. Nonsignificant GO terms within the hierarchical tree are shown as white boxes. Branches of the GO hierarchical tree without significantly enriched GO terms are not shown. Arrows represent connections between different GO terms. Red arrows represent relationships between two enriched GO terms, black solid arrows represent relationships between enriched and unenriched terms and black dashed arrows represent relationships between two unenriched GO terms.

**Figure 12:**
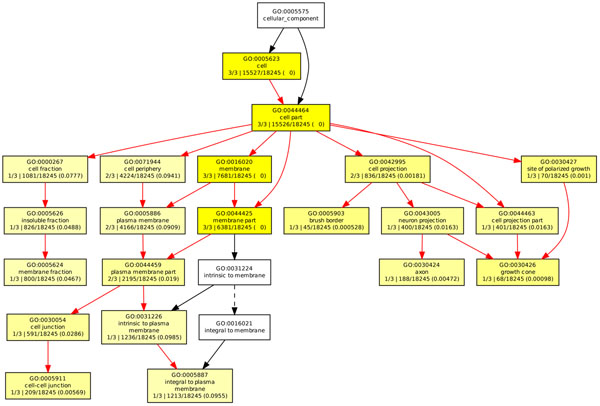
**GOEAST analysis in cellular component category of Hirschsprung study.** The GOEAST graphical output of enriched GO terms in the cellular component category for our selected gene network of Hirschsprung study. Boxes represent GO terms, labeled by its GO ID, term definition, and detailed information, organized as “*q*/*m*|*t*/*k*(*p* – *value*)”, where *q* is the count of genes associated with the listed GOID (directly or indirectly) in our dataset, *m* is the count of genes associated with the listed GOID (directly or indirectly) on the chosen platform, *k* is the total number of genes in our dataset, *t* is the total number of genes on the chosen platform, *p*-value is of the significance for the enrichment in the dataset of the listed GOID under hypergeometric distribution. Significantly enriched GO terms are marked yellow. The degree of color saturation of each node is positively correlated with the enrichment significance of the corresponding GO term. Nonsignificant GO terms within the hierarchical tree are shown as white boxes. Branches of the GO hierarchical tree without significantly enriched GO terms are not shown. Arrows represent connections between different GO terms. Red arrows represent relationships between two enriched GO terms, black solid arrows represent relationships between enriched and unenriched terms and black dashed arrows represent relationships between two unenriched GO terms.

For the gene network shown in Figure 9, the same procedure of enrichment analysis using GOEAST was done, see Figures 10,11,12. Table 9 shows a detailed description of GO terms with a significant *P* < 0*.*0001, which can provide an inner functions of our selected genes.

**Table 9:** Gene Ontology terms significant associated with selected genes (*P* < 0.0001) of Hirschsprung study.

GO Category	GO ID	GO Annotation	Genes	*P*-value
Biological Process	GO:0050772	positive regulation of axonogenesis	DSCAM, TIAM1	0.000000736
Biological Process	GO:0031346	positive regulation of cell projection organization	DSCAM, TIAM1	0.000005400
Biological Process	GO:0048013	ephrin receptor signaling pathway	TIAM1	0.000024800
Biological Process	GO:0031344	regulation of cell projection organization	DSCAM, TIAM1	0.000030600
Biological Process	GO:0009886	post-embryonic morphogenesis	DSCAM	0.000038400
Biological Process	GO:0048841	regulation of axon extension involved in axon guidance	DSCAM	0.000045900
Molecular Function	GO:0046875	ephrin receptor binding	TIAM1	0.000053600

## Conclusions

In this paper, we use the method of nearest shrunken centroid for gene expression data, and apply it to tackle copy number variation data determined from genome-wide SNP arrays. The method can be implemented on a personal computer very efficiently. The relevant SNPs are selected for disease data. Experimental results are reported to show the effectiveness of our method. In particular, we find some SNPs that contain in some genes which are relevant to a particular disease. Based on the SNP and gene networks, we can find out some unknown relationships between their corresponding genes, which can be considered as an extension of existing GO knowledge. The existing Gene Ontology enrichment analysis tool also suggests that our selected genes are associated to some molecular function and biological process. In the future, we will study the following problems. Detailed biological analysis of CNVs determined from other genome-wide SNP data sets will be studied. Statistical and association study of selected SNPs can be carried out.

## Competing interests

The authors declare that they have no competing interests.

## Authors contributions

MN designed this study and developed the new algorithm. YL designed this study, coded the program, ran the experiments and wrote the manuscript. YF designed this study.
